# A Pleasant Substitute for Epsom Salts as a Purgative

**Published:** 1847-10

**Authors:** 


*A Pleasant Substitute for Epsom Salts as a purgative.*—M. Garot recommends the following formula for the preparation of tasteless purgative salts (citrate of magnesia):

| | |
|---|---|
| Carbonate of magnesia, - - - - | 15 parts. |
| Citric acid, - - - - - | 21 to 22 " |
| Aromatic syrup, - - - - - - | 60 " |
| Water, - - - - - - - - | 300 " |

The citric acid is separately dissolved and added to the carbonate of magnesia diffused in water.

As thus prepared it is not effervescing, but it is easily rendered so by adding only half the quantity of acid, and reserving the addition of the other half until the dose is taken. The above proportions in grains would constitute a dose.

Dr. Pereira long since suggested the use of citrate of magnesia in nearly similar proportions. He found that one scruple of crystalized citric acid saturated about fourteen grains of light or heavy carbonate of magnesia.—*Lond. Med. Gaz.*

